# Rheumatoid Arthritis

**Published:** 1901-10-26

**Authors:** 


### Hospital Clinics and Medical Progress.

### RHEUMATOID ARTHRITIS.

When years ago it was shown that in addition to those cases of arthritis which were definitely rheumatic and those which were of gouty origin there were many joint affections which had nothing to do with either of these diseases, and when these cases were erected into a special class to themselves under the name of rheumatoid arthritis, although the name chosen was perhaps unfortunate, a great advance was made. All differentiation of diseases leads to exactitude, and the question arises whether the time has not now come when some still more minute subdivision of these cases may be possible. For years back the position of affairs has been somewhat as follows:—Gout is a sufficiently definite pathological entity and may be put on one side. Then there are a large number of cases of arthritis which appear to be due to bacteria or their products. It is now usual to place acute rheumatism in this class, and in addition to it we must include the joint lesions of pyæmia, gonorrhœa, syphilis, and those which occur in the course of the infective fevers. Then we come to the arthropathies which are due to nervous diseases, such as those which occur in connection with locomotor ataxia, and others which are directly due to injury. But when we have separated all these there remains a considerable residue of cases which have been variously described as chronic rheumatism, rheumatic gout, and rheumatoid or osteo-arthritis. Dr. Archibald Garrod raises the question whether the cases commonly grouped under the names of rheumatoid or osteo-arthritis are really examples of a single malady, and whether we may not now proceed a step further in differentiation and establish out of this hitherto unsorted residue at least two distinct diseases. By careful clinical observation of such cases he is able to throw a large proportion of them into one or other of two groups, one consisting of what he calls the "fusiform" cases and the other of cases in which the malady assumes the "nodular" form; but he is far from suggesting that these two categories embrace all the cases. He goes on to say that if it be true that under the name of rheumatoid or osteo-arthritis we have been in the habit of confusing together two or more distinct diseases it is obvious that no single pathological theory will suffice to cover them all. Other observers, however, are not inclined to seek for advance in the direction of further differentiation of the residue on a pathological or anatomical basis. Indeed they suggest that what we rather have to do is to search carefully for an etiological basis of classification, for causes rather than for anatomical distinctions. Dr. Cave of Bath, for example, holds that various anatomical forms of disease may result from the same infective agent, and conversely that various infective agents may produce the same anatomical form. The anatomical characters of some forms of traumatic, or gouty, or gonococcal arthritis, for example, are not to be distinguished from those of the most typical forms of "arthritis deformans." From the clinical side also the same thesis may be maintained, and looking at the matter from the standpoint of therapeutics it is important to recognise that we must fix our attention on the etiological conditions and carry back our researches to the very beginning of each case. When once started the malady may be self-propagating. We want to discover how it began; what was the microbial or toxic or other cause by which the disease was originally set up. The cavities of the nose and ear and their accessory sinuses, the buccal cavity, the whole extent of the alimentary canal and its diverticula, the skin, the respiratory tract, the genital and urinary organs, must all be inquired into and, if necessary, scrutinised in our search for a toxic or other cause of the disease. According to this view we must not form our judgment merely from the anatomical characters which may be present. Evidently the last word is far from having been said on this important subject. It is all very well to say that all sorts of causes may produce the same effects; but that is exactly one of the points which are at issue, and we cannot but wish that Dr. Garrod may be able by his careful observations to go still further than he has done in the differentiation of the several groups of cases—so far, indeed, as to attach them each to its own separate etiological condition. It is a great advance to have reached the point at which we have surely now arrived, namely, that in the presence of a case of arthritis we must not be content merely to ask ourselves, is this gout? or is it rheumatism? or is it rheumatoid arthritis? but must go further and inquire of what pathological condition, microbial, septic, or dystrophic, may it be the symptom. But it would be a greater advance still, and would be worth anything from a clinical and practical point of view, if Dr. Garrod could carry still further his differentiations, and could connect his various groups of arthritis with the several causes by which they are provoked. This, however, is, we fear, a long way off.

